# Small fiber neuropathy in the post‐COVID condition and Myalgic Encephalomyelitis/Chronic Fatigue Syndrome: Clinical significance and diagnostic challenges

**DOI:** 10.1111/ene.70016

**Published:** 2025-01-31

**Authors:** Naiara Azcue, Sara Teijeira‐Portas, Beatriz Tijero‐Merino, Marian Acera, Tamara Fernández‐Valle, Unai Ayala, Maitane Barrenechea, Ane Murueta‐Goyena, Jose Vicente Lafuente, Adolfo Lopez de Munain, Guillermo Ruiz‐Irastorza, Daniel Martín‐Iglesias, Iñigo Gabilondo, Juan Carlos Gómez‐Esteban, Rocio Del Pino

**Affiliations:** ^1^ Neurodegenerative Diseases Group Biobizkaia Health Research Institute Barakaldo Spain; ^2^ Department of Neurology Cruces University Hospital‐OSAKIDETZA Barakaldo Spain; ^3^ Biomedical Engineering Department, Faculty of Engineering (MU‐ENG) Mondragon Unibertsitatea Mondragón Spain; ^4^ Department of Neurosciences University of the Basque Country UPV/EHU Leioa Spain; ^5^ Department of Neurology Donostia University Hospital‐OSAKIDETZA San Sebastián Spain; ^6^ Department of Neurosciences Biogipuzkoa Health Research Institute San Sebastián Spain; ^7^ CIBERNED‐CIBER, Institute Carlos III Madrid Spain; ^8^ Autoimmune Diseases Research Unit, Biobizkaia Health Research Institute Barakaldo Spain; ^9^ Department of Autoimmune Diseases Cruces University Hospital‐OSAKIDETZA Barakaldo Spain; ^10^ The Basque Foundation for Science, IKERBASQUE Bilbao Spain

**Keywords:** Myalgic Encephalomyelitis/Chronic Fatigue Syndrome, post‐COVID condition, small fiber neuropathy

## Abstract

**Background:**

Patients with post‐COVID condition (PCC) and Myalgic Encephalomyelitis/Chronic Fatigue Syndrome (ME/CFS) experience symptoms potentially associated with small fiber neuropathy (SFN).

**Methods:**

A sample of 90 participants, comprising 30 PCC patients, 30 ME/CFS patients, and 30 healthy controls (HC), matched by sex and age, was assessed. Neuropathic, autonomic, and fatigue symptoms were measured with TaskForce Monitor, the Sudoscan, heat and cold evoked potentials, In Vivo Corneal Confocal Microscopy (IVCCM), and specialized questionaries.

**Results:**

PCC and ME/CFS patients demonstrated significantly higher levels of autonomic symptoms (*H* = 39.89, *p* < 0.001), neuropathic symptoms (*H* = 48.94, *p* < 0.001), and fatigue (*H* = 49.29, *p* < 0.001) compared to HC. Quantitative sensory testing revealed significant differences in heat detection thresholds between PCC patients and HC (*F* = 4.82; *p* < 0.01). Regarding corneal small fiber tortuosity, there were statistically significant differences between patients and HC (*F* = 6.80; *p* < 0.01), indicating pathological responses in patients. Small fiber tortuosity in IVCCM was identified as the main discriminator between patients and HC (AUC = 0.720; *p* < 0.01).

**Conclusion:**

PCC and ME/CFS patients demonstrated sensory SFN, as evidenced by impaired heat detection and increased tortuosity of small fibers in the central corneal subbasal plexus. The findings underscore the importance of a multimodal approach to comprehensively detect and characterize SFN. This study provides valuable scientific insights into the neuropathic manifestations associated with these conditions.

## INTRODUCTION

Post‐COVID condition (PCC) and Myalgic Encephalomyelitis/Chronic Fatigue Syndrome (ME/CFS) are syndromes marked by severe fatigue, cognitive deficits, myalgia, polyarthralgia, unrefreshing sleep, headaches, and tachycardia [1, 2, 3, 4]. Additionally, these patients often exhibit autonomic and neuropathic symptoms, including orthostatic intolerance, tingling, or limb pain, which may originate from disruptions in the peripheral nervous system [5, 6]. Approximately 70%–90% of the peripheral nervous system consists of fibers with minimal (Aδ fibers) or no myelination (C‐type fibers) [7]. Dysfunction or damage to these fibers results in small fiber neuropathy (SFN).

SFN can manifest as sensory, autonomic, or mixed patterns. Sensory neuropathy gives rise to sensations of burning, tingling, pricking, and even pain [8]. Likewise, autonomic neuropathy can lead to symptoms include dry eyes, dry mouth, orthostatic dizziness, palpitations, and gastrointestinal or genitourinary disturbances [9]. The most prevalent form of SFN is length‐dependent [10], wherein the longest fibers are the first to succumb to damage. Consequently, initial symptoms typically manifest in the lower limbs before progressing to the upper limbs [10]. This type of neuropathy is commonly associated with metabolic conditions such as diabetes [9, 10]. Alternatively, neuropathy can be non‐length‐dependent, resulting in “patchy,” non‐uniform patterns [10], and it is often associated with infectious diseases, exposure to toxic agents, or autoimmune etiologies [8, 10].

Various technologies are available for assessing the presence of neuropathy, with one of the most commonly employed being skin biopsy, which evaluates small nerve fiber density in the skin. However, less invasive methodologies have gained popularity for their diagnostic and monitoring utility [7], such as the Sudoscan (Impeto Medical© Paris) [11, 12], which measures electrochemical skin conductance (ESC); heat and cold evoked potentials; quantitative sensory testing (QST) [13]; and in vivo corneal confocal microscopy (IVCCM) [7, 14, 15].

This study aimed to investigate SFN in patients with ME/CFS and PCC by evaluating small fiber electrochemical responses, nerve reaction times to temperature stimuli, and corneal small fiber morphology. Additionally, it sought to differentiate neuropathy subtypes, identify associated symptoms, and determine the most effective diagnostic techniques for SFN. To enhance these assessments, pupillary responses and hemodynamic autonomic functions were analyzed to investigate autonomic nervous system (ANS) dysfunction and its potential association with SFN in these patient populations.

## MATERIALS AND METHODS

### Participants and demographic data

Thirty participants with PCC, 30 with ME/CFS, and 30 healthy controls (HC) were recruited through the Neurology Department at the Cruces University Hospital. Participants were carefully matched by age and sex to ensure group comparability. Demographic data, neuropathic and autonomic symptoms, and fatigue levels were recorded.

Inclusion criteria required adults aged 18 to 85 with adequate communication skills and willingness to participate. Exclusion criteria included pregnancy or lactation, severe trauma, substance abuse, severe cardiac conditions, structural brain pathology, metabolic or autoimmune diseases, oncological conditions, prior eye surgery, or any concomitant disease that could potentially influence the results.

Specifically, patients diagnosed with ME/CFS were required to either have a prior diagnosis or meet the Fukuda et al. criteria [3] at the time of evaluation. These criteria require the patient to have marked fatigue for at least 6 months that is not explained by the physical or mental effort. The fatigue must be accompanied by at least four of the following symptoms: unrefreshing sleep, impaired memory, post‐exertion malaise, arthralgia, headache, sore throat, and tender lymph nodes [3]. Additionally, all ME/CFS patients included in the study reported post‐exertional malaise.

Those diagnosed with PCC adhered to the criteria outlined in the NICE guidelines, involving signs and symptoms persisting for more than 12 weeks after COVID‐19 infection, not attributable to an alternative diagnosis [4]. For acute COVID‐19 diagnosis, valid methods included a positive nasal PCR test, detection of IgG and/or IgM antibodies against SARS‐CoV‐2, or a medical report supporting the diagnosis. Exclusion criteria for this group comprised respiratory symptoms persisting for 12 weeks post‐infection, admission to an intensive care unit, severe bilateral pneumonia, or other manifestations necessitating hospitalization.

The study protocol received approval from the Basque Drug Research Ethics Committee [Comité de Ética de la Investigación con Medicamentos de Euskadi (CEIm‐E) (PI2020210)]. Prior to participation, all individuals provided written informed consent, adhering to the principles of the Declaration of Helsinki.

### Small fiber function assessment

The functioning of nerve signals from the ANS that regulate sweat production was non‐invasively quantified using the Sudoscan® device. It assesses ESC in the palms and soles, and gauge sweat gland function by analyzing sweat chloride concentrations through reverse iontophoresis and chronoamperometry. ESC results are expressed in microSiemens (μS) [11].

The sensory small fibers' function was assessed with the TSA‐2 device (Medoc Advanced Medical Systems, Ramat Yishai, Israel), encompassing Contact Heat Evoked Potentials (CHEPs), Cold Evoked Potentials (CEPs), and QST. For CHEPs, 15 stimuli of 55°C were applied every 30–45 s, while CEPs involved 15 stimuli of 9°C at similar intervals. QST, a psychosensory test, measured sensory thresholds for pain, as well as hot and cold temperature sensations.

The subbasal plexus of the cornea was examined using the IVCCM module of HRT‐3/RCM of Heidelberg Engineering Inc.©. The laser wavelength was 670 nm, the magnification was ×800, and the resolution was 1 μm. Before the examination, both eyes were anesthetized, carbomer eye drops were applied to the surface of the objective lens, and a sterile disposable cover was placed. Each cornea was scanned in the subbasal nerve plexus central region from the right and left eyes. The region in which the fibers were observed at 90° was considered the central cornea. The field of view for each image was a 400 × 400 μm area. The 15 most representative images of the central cornea were selected for each patient. All procedures were performed by the same specialist. Images were analyzed with ACCMetrics software (University of Manchester) [16, 17, 18]. The nerve metrics were corneal nerve fiber density (CNFD), corneal nerve branch density (CNBD), corneal nerve fiber length (CNFL), corneal nerve fiber total branch density (CTBD), corneal nerve fiber area (CNFA), corneal nerve fiber width (CNFW), and fractal dimension. The tortuosity of the nerve fibers was determined by analyzing changes in fiber angles using a custom‐made algorithm developed in MATLAB. The average of the two eyes was calculated for each of the variables. The number of dendritic cells of the cornea's subbasal plexus was also analyzed. Five non‐overlapping images of the central cornea were taken for this analysis, an average was not calculated; instead, a total count was performed. Dendritic cells were counted with the CCMetrics software (University of Manchester).

### ANS assessment

For the assessment of the ANS, a pupillometer (PLR‐3000, Neuroptics) was employed. Pupillometry, a diagnostic test, facilitates the measurement of pupil size and its response to specific stimuli. In our study, participants underwent a 5‐minute dark adaptation period before testing to establish baseline pupillary conditions. Subsequently, pupillary changes were recorded over a 5.01‐s duration following a white light stimulus with a pulse duration of 800 ms. The initial and final diameter of each pupil, latency of pupillary response, maximum contraction speed, average contraction speed, average dilation speed, and the time (in seconds) required for the pupil to recover 75% of its initial diameter were meticulously documented.

A complete ANS assessment was performed for every patient. Non‐invasive quantitative measures for hemodynamic autonomic function were determined with a Task Force Monitor (CNSystems, Graz, Austria) following standard procedures for quantitative autonomic testing [19]. Heart rate (HR) and blood pressure (BP) variability were continuously monitored during the hemodynamic autonomic evaluation. Parasympathetic function was analyzed using the deep breathing technique, and sympathetic function was assessed with the Valsalva maneuver. Finally, an 11‐min tilt table test was performed. All autonomic function tests were carried out by experienced neurologists.

### Symptom assessment

Autonomic symptoms were assessed using the Composite Autonomic Symptom Score (COMPASS‐31), which evaluates patient‐reported manifestations across six dimensions of autonomic function: orthostatic intolerance, sudomotor, vasomotor, gastrointestinal, bladder, and pupillomotor domains. Symptoms related to Small Fiber Neuropathy (SFN) were evaluated using the Small Fiber Neuropathy Screening List (SFNSL), which consists of 21 questions. Fatigue levels were assessed using the Modified Fatigue Impact Scale (MFIS), which quantifies the impact of fatigue on daily functioning using 21 items.

### Statistical analysis

Statistical analyses were conducted using IBM SPSS Statistics for Windows, version 26.0 (IBM SPSS, Armonk, NY, USA). Assumptions of normality and homogeneity of variances were assessed for all variables.

Group differences for continuous and categorical variables were analyzed using the Kruskal–Wallis test and Chi‐square test, respectively. Kruskal–Wallis statistics were adjusted with Bonferroni corrections for multiple comparisons. The Mann–Whitney *U* test was employed to analyze differences in disease duration between the PCC and ME/CFS groups. An ANOVA was performed to determine differences between the three groups in IVCCM measures, Sudoscan, QST, CHEPs, and CEPs. For IVCCM measures, the use of contact lenses was also included as a covariate. Receiver Operating Characteristic (ROC) curve analysis was performed to identify the variables that best‐discriminated patients from HC. Finally, Spearman bivariate correlations were calculated to analyze the relationships among small fiber assessment parameters. The level of statistical significance was set at *p* < 0.05 (two‐tailed).

## RESULTS

### Demographic and Clinical Data

Participants were matched by age (42.89 ± 8.47 years) and sex (90.0% women). ME/CFS patients had a significantly longer disease duration than PCC patients. Both groups exhibited elevated fatigue levels compared to HC (*H* = 49.29, *p* < 0.001), with no differences between ME/CFS and PCC patients (Table 1).

**TABLE 1:** Clinical data.

	PCC M (SD)	ME/CFS M (SD)	HC M (SD)	Statistics	Bonferroni (*p*)	
					PCC vs HC	ME/CFS vs HC
Age, years	43.96 (8.75)	42.96 (8.19)	41.73 (8.59)	*F* = 0.52		
Female, *n* (%)	27 (90.00)	27 (90.00)	27 (90.00)			
Disease duration, months	20.40 (8.88)	66.60 (64.82)		*U* = 8.08**		
COMPASS 31	21.10 (10.04)	24.03 (9.17)	16.00 (4.12)	*H* = 39.89***	≤0.001	≤0.001
SFNSL	26.03 (11.55)	34.25 (15.54)	1.20 (3.06)	*H* = 48.94***	≤0.001	≤0.001
MFIS	66.73 (15.70)	65.83 (11.15)	8.00 (10.50)	*H* = 49.29***	≤0.001	≤0.001
Contact lenses, *n* (%)	0 (0.00%)	2 (6.67)	2 (6.67)	*χ* ^2^ = 2.09		

Abbreviations: COMPASS, The Composite Autonomic Symptom Score; HC, healthy controls; ME/CFS, Myalgic Encephalomyelitis/Chronic Fatigue Syndrome; MFIS, Modified Fatigue Impact Scale; PCC, post‐COVID condition; SFNSL, Small Fiber Neuropathy Screening List.

*p ≤ 0.05; **p ≤ 0.01; ***p ≤ 0.001.

### Autonomic Symptoms

The COMPASS‐31 revealed significant differences between patients and HC (*F* = 39.87, *p* < 0.001) (Table 1). ME/CFS exhibited the lowest ESC (μS) in palms (64.75 ± 16.64), followed by PCC patients (69.17 ± 13.07), and HC (71.41 ± 11.76). No statistically significant differences in autonomic small fiber function were found between groups.

### Sensory Symptoms

**FIGURE 1:**
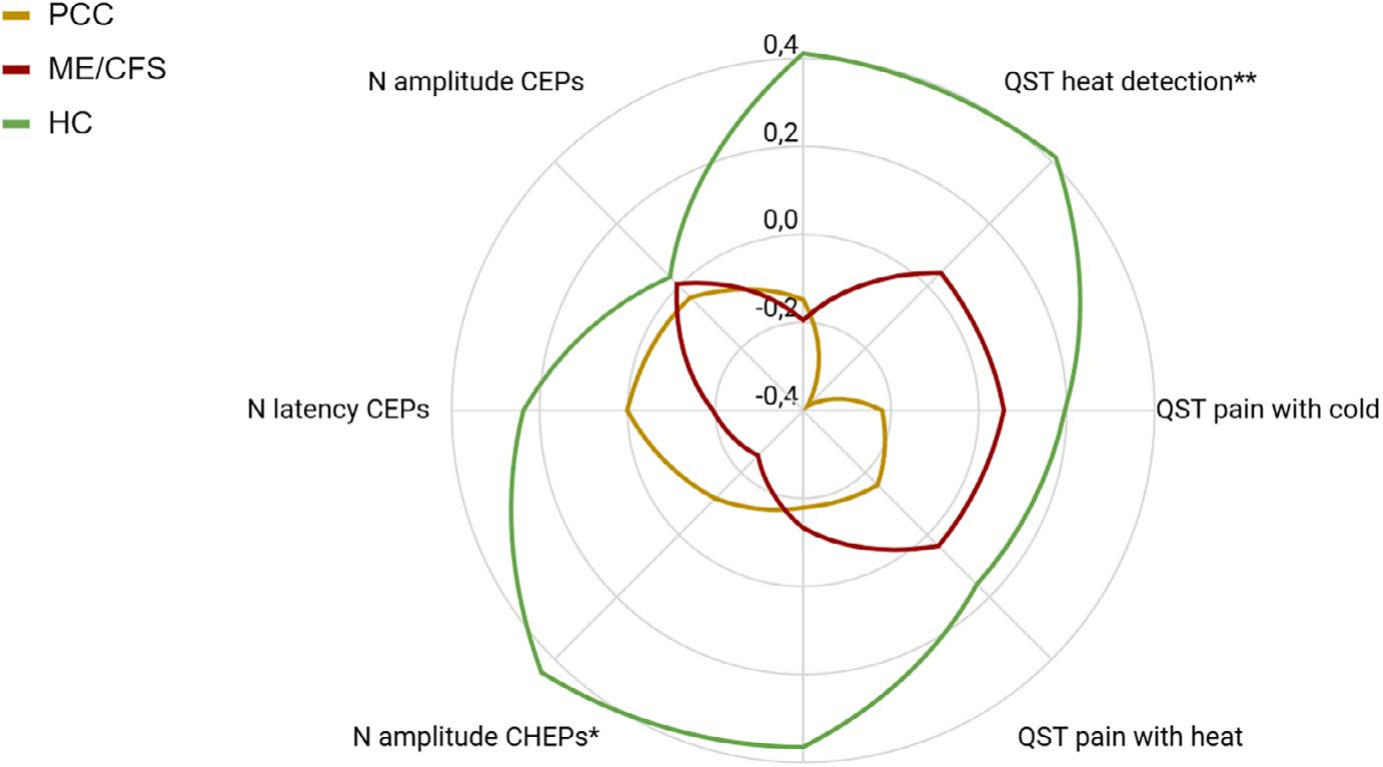
QST, CHEPs, and CEPs. Comparison of the results between the three groups for the QST, CHEPs, and CEPs tests. Data is shown in z‐scores, a higher value means a better response. *p ≤ 0.05; **p ≤ 0.01. CEPs, cold evoked potentials; CHEPs, Contact heat evoked potentials; HC, Healthy controls; ME/CFS, Myalgic Encephalomyelitis/Chronic Fatigue Syndrome; QST, Quantitative sensory testing.

The SFNSL showed statistically significant differences between the three groups. ME/CFS group presented more SFN symptoms, followed by PCC group, and finally HC (Table 1). Heat detection thresholds were significantly impaired in PCC compared to HC, whereas ME/CFS patients exhibited lower N amplitude in CHEPs compared to HC (Table 2; Figure 1).

**TABLE 2:** Sensory small fiber assessment.

	PCC M (SD)	ME/CFS M (SD)	HC M (SD)	ANOVA (F)	Bonferroni (*p*)	
					PCC vs HC	ME/CFS vs HC
Sudoscan ESC						
Feet (μS)	69.33 (14.44)	72.28 (11.30)	70.33 (16.05)	0.33		
Hands (μS)	69.17 (13.07)	64.75 (16.64)	71.41 (11.76)	1.54		
QST						
Heat detection	36.96 (3.22)	35.91 (1.72)	34.99 (1.65)	4.82**	0.008	
Cold detection	25.50 (5.88)	25.26 (6.09)	28.42 (1.64)	3.14*		
Pain with heat	43.04 (4.61)	41.74 (4.45)	41.07 (4.99)	1.27		
Pain with cold	13.26 (10.42)	15.12 (8.51)	16.31 (9.70)	0.71		
Contact evoked potentials						
Heat						
N latency	0.64 (0.19)	0.63 (0.18)	0.54 (0.15)	2.47		
N amplitude	−9.93 (5.45)	−9.03 (4.67)	−13.60 (8.61)	3.85*		0.028
Cold						
N latency	0.34 (0.09)	0.36 (0.10)	0.32 (0.06)	1.29		
N amplitude	−8.96 (5.11)	−9.28 (9.64)	−9.4 (6.38)	0.26		
IVCCM						
CNFD	30.15 (6.63)	30.73 (6.16)	32.37 (5.42)	1.07		
CNBD	39.70 (14.84)	44.79 (19.94)	42.23 (17.17)	0.64		
CNFL	17.17 (3.47)	17.28 (2.94)	18.11 (3.27)	0.75		
CTBD	56.12 (23.77)	56.60 (26.51)	57.12 (24.38)	0.01		
CNFA	0.07 (0.02)	0.006 (0.002)	0.07 (0.02)	0.09		
CNFW	0.02 (0.00)	0.02 (0.00)	0.02 (0.00)	0.94		
Fractal dimension	1.50 (0.02)	1.51 (0.05)	1.50 (0.01)	0.78		
Tortuosity	1.12 (0.02)	1.13 (0.01)	1.11 (0.01)	6.80**	0.012	0.003
Pupillometry						
Mean of both eyes						
PLR Diameter Init	6.21 (0.74)	6.36 (0.65)	6.52 (0.79)	0.86		
PLR Diameter End	3.79 (0.53)	3.90 (0.65)	4.07 (0.71)	0.97		
PLR Latency	0.22 (0.01)	0.22 (0.02)	0.22 (0.02)	0.39		
PLR Constriction Velocity	−2.98 (0.38)	−3.02 (0.44)	−2.87 (0.32)	0.75		
PLR Dilation Velocity	1.12 (0.20)	1.17 (0.22)	2.77 (0.36)	0.86		

*Note*: ANCOVA was made using gender and age as covariates.

Abbreviations: CNBD, corneal nerve branch density; CNFA, corneal nerve fiber area; CNFD, corneal nerve fiber density; CNFL, corneal nerve fiber length; CNFW, corneal nerve fiber width; CTBD, corneal nerve fiber total branch density; EMM, estimated marginal means; ESC is shown microsiemens; latency is shown in milliseconds, and amplitude is shown in microvolts; ESC, electrochemical skin conductance; HC, healthy controls; Init., initial; ME/CFS, Myalgic Encephalomyelitis/Chronic Fatigue Syndrome; PCC, post‐COVID condition; PLR, pupil light reflex; QST, quantitative sensory testing; T75, time (in seconds) that it takes the pupil to recover 75% of the initial diameter were recorded.

*p ≤ 0.05; **p ≤ 0.01

### In Vivo Corneal Confocal Microscopy

**FIGURE 2:**
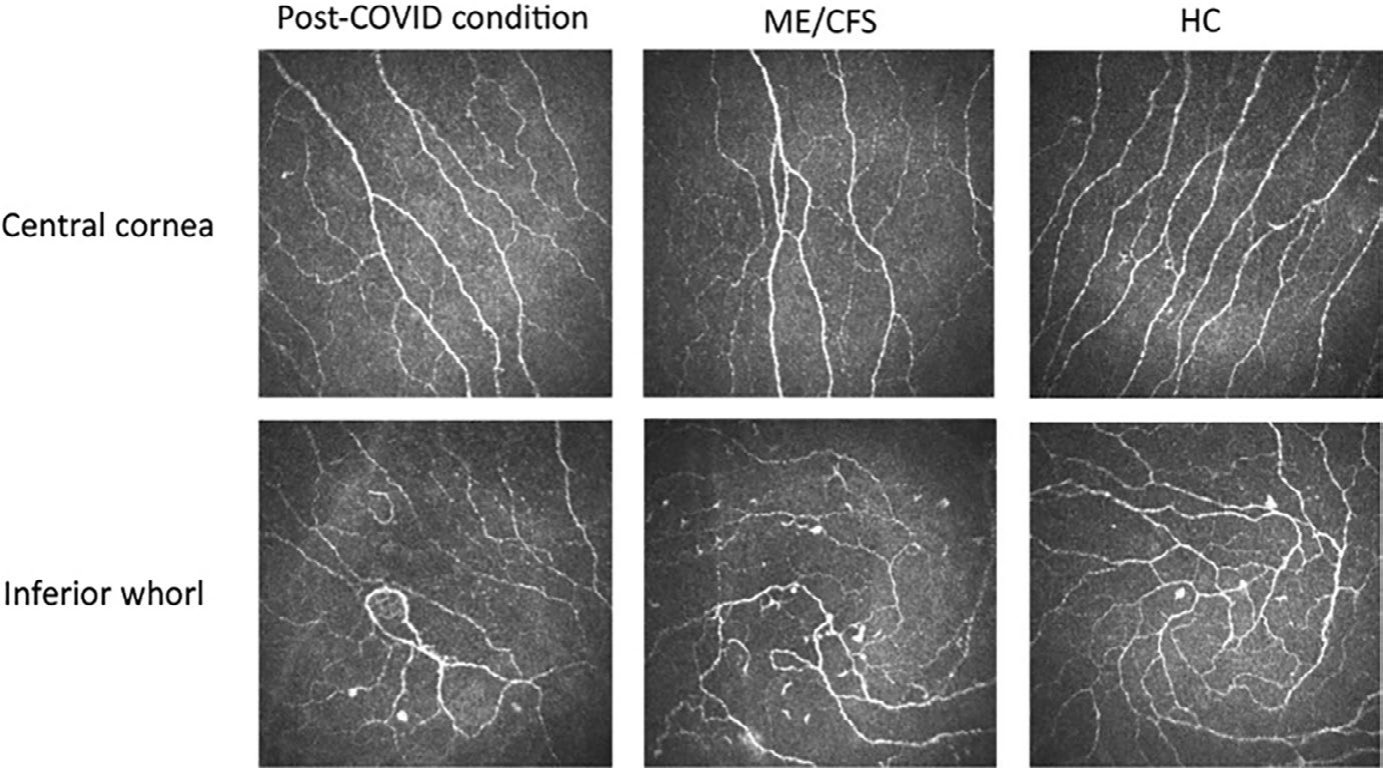
Cornea subbasal plexus. Images acquired with In Vivo Corneal Confocal Microscopy of the central and inferior area of the cornea. The images are the most representative ones for every group. HC, Healthy controls; ME/CFS, Myalgic Encephalomyelitis/Chronic Fatigue Syndrome.

IVCCM images showed slightly different patterns between patients and HC (Figure 2). However, the analysis of dendritic cells did not reveal any statistically significant differences in cell quantity between patients and HC.

**FIGURE 3:**
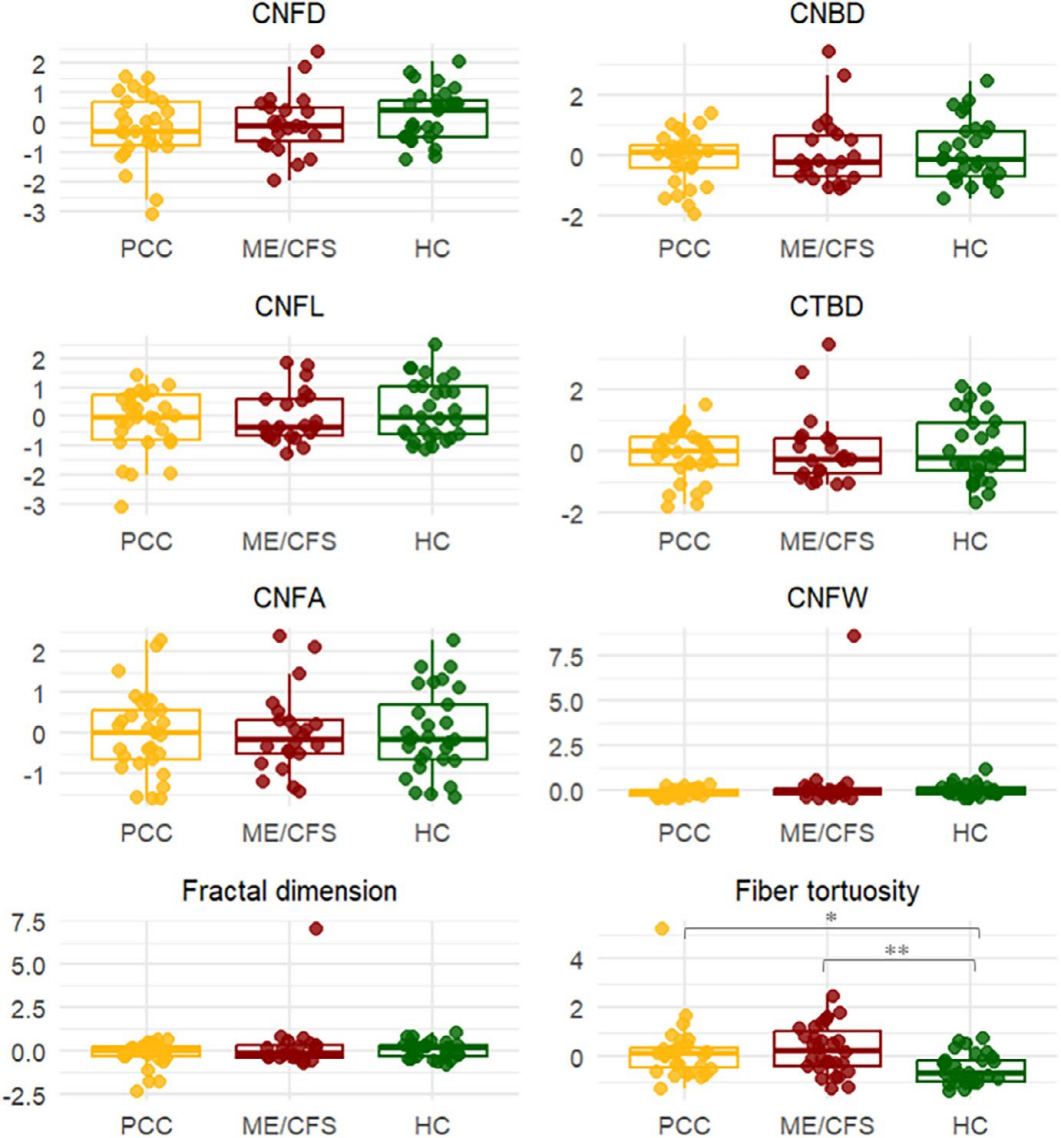
In Vivo Corneal Confocal Microscopy in PCC patients, ME/CFS and HC. Comparison of the measurements obtained with ACCMetrics in the images of the central area of the cornea using In Vivo Corneal Confocal Microscopy. Values are shown in z‐scores. CNBD, Corneal nerve branch density; CNFD, Corneal nerve fiber density; CNFL, Corneal nerve fiber length; CTBD, Corneal nerve fiber total branch density; CNFA, Corneal nerve fiber area; CNFW, Corneal nerve fiber width; HC, Healthy controls; ME/CFS, Myalgic Encephalomyelitis/Chronic Fatigue Syndrome.

Fiber density, branch density, total fiber and branch density, fiber length, total fiber area, fiber width, and the fractal dimension of the central cornea were analyzed. There were no statistically significant differences in any of them. Although no statistically significant differences were found, values were higher for fiber density and length in HC (Table 2; Figure 3).

In addition to the variables analyzed by ACCMetrics, the tortuosity of small fibers in the central cornea was investigated. Statistically significant differences were found between groups (*F* = 6.80, *p* = 0.002), specifically between HC and ME/CFS patients (*p* = 0.003) and between HC and PCC (*p* = 0.012), with worse tortuosity observed in patients than in HC (Table 2; Figure 3).

ROC curves were used to determine the discriminatory ability of variables with significant differences between patients and HC. The variable most effective in discriminating between HC and PCC patients was the tortuosity of corneal subbasal plexus nerve fibers (AUC = 0.741; *p* = 0.003), followed by heat detection (N latency) in CHEPs (AUC = 0.668; *p* = 0.038). In ME/CFS patients, the variable that best discriminated between them and HC was also fiber tortuosity (AUC = 0.690; *p* = 0.032).

Taking both PCC patients and ME/CFS patients as a whole group, ROC analysis revealed small fiber tortuosity as the main discriminator (AUC = 0.720; *p* = 0.003), followed by CHEPs N latency (AUC = 0.656; *p* = 0.032).

### ANS Assessment

The pupillary response to a light stimulus was analyzed to assess the sympathetic and parasympathetic responses of the ANS (Table 2). No statistically significant differences were found between any of the three groups in the initial and final diameter of pupils, latency of pupillary response, maximum contraction speed, average contraction speed, average dilation speed, or in the time (in seconds) for the pupil to recover 75% of the initial diameter.

No statistically significant differences were found in the deep breathing technique or the Valsalva maneuver among the three groups analyzed. However, differences were found in the response to the Tilt test between the ME/CFS patients and the other two groups (*F* = 8.41, *p* ≤ 0.001), with 30% of the cases meeting the criteria for Postural Orthostatic Tachycardia Syndrome (POTS). None of the PCC patients or HC met the criteria for this syndrome.

### Correlations

Correlations between ESC (μS), small fiber reaction time (ms) to temperature changes, fiber morphology, pupilar responses (ms), COMPASS‐31, SFNSL, MFIS, and disease duration were examined for each group.

In PCC patients, more symptoms in the SNFSL correlated with higher levels of fatigue (Rho = −0.41; *p* = 0.029), and more autonomic symptoms (Rho = 0.48; *p* = 0.010). Better cold detection in the QST was also associated with better heat detection in this test (Rho = 0.59; *p* ≤ 0.001). In IVCCM analysis, a lower fiber density was found to correlate with an increased number of autonomic symptoms (Rho = −0.42; *p* = 0.024). Longer disease duration in this group correlated with worse cold detection in the QST (Rho = −0.39; *p* = 0.029), and increased pupillary dilation velocity was correlated with more autonomic symptoms, as measured by the COMPASS‐31 (Rho = 0.56; *p* = 0.003).

In ME/CFS patients, more neuropathic symptoms were associated with more autonomic symptoms (Rho = 0.47; *p* = 0.022), higher levels of fatigue (Rho = 0.60; *p* = 0.002), and a longer disease duration (Rho = 0.50; *p* = 0.034). As observed in PCC patients, better heat detection in the QST was also correlated with better cold detection in this test (Rho = 0.50; *p* = 0.004). Higher ESC in the hands correlated with a shorter disease duration (Rho = −0.48; *p* = 0.032), and higher ESC in the feet was associated with greater small fiber density (Rho = 0.38; *p* = 0.043). Longer blood pressure recovery time during the Valsalva maneuver was associated with lower ESC values in the feet (Rho = −0.39, *p* = 0.042) and hands (Rho = −0.39, *p* = 0.039). Better ESC in hands correlated with lower levels of fatigue (Rho = −0.41, *p* = 0.033).

## DISCUSSION

The primary objective of this study was to ascertain the presence of sensory or autonomic SFN in individuals with PCC and ME/CFS, as compared to HC. Additionally, the study aimed to determine the most appropriate technique for assessing neuropathy in these patient groups.

Both PCC and ME/CFS patients exhibited impaired heat detection. However, in contrast to findings from previous studies [7, 20, 21], no significant differences in small fiber density measures were observed between the groups. A more detailed analysis of corneal subbasal plexus small fibers, considering their tortuosity, suggested an emerging SFN [22]. The inconsistency in results across studies, including the present one, analyzing IVCCM variables in these pathologies might suggest that SFN is not an inherent characteristic of these diseases. Instead, the underlying process may be inflammatory rather than neurodegenerative, and produce neuroapraxia rather than denervation (neurotmesis). In neurodegenerative conditions like amyloidosis, Fabry disease [23, 24], or advanced diabetes [25], where the central cornea's small fibers are affected, a progressive denervation is typically observed over time. However, signs of SFN in IVCCM were not linked to the duration of the disease in our patients.

Although the differences were not statistically significant, HC exhibited higher fiber density and length values. This pattern, while not “abnormal” by traditional clinical cutoffs, may still be relevant. In chronic conditions such as ME/CFS or other infection‐associated illnesses, it is common to observe borderline abnormal findings that are not severe enough to cross conventional diagnostic thresholds. This has been noted in studies of cortisol levels, where dysregulation may exist without breaching abnormal ranges [26, 27]. This borderline pattern could indicate an underlying dysregulation that standard metrics fail to capture fully.

The limited correlations found across these techniques in all patients may indicate a “patchy” manifestation pattern of SFN, making it less consistent in various body parts. This “patchy” distribution of small fiber neuropathy can present a challenge for neurologists, as it may not be as easily recognized as the more typical length‐dependent form of SFN. Post‐infectious patients, including those with PCC or ME/CFS, may not exhibit a clear gradient of nerve dysfunction from distal to proximal regions but rather isolated or non‐uniform involvement of small fibers. This variability highlights the need for clinicians to employ a range of diagnostic techniques, such as QST, IVCCM, CHEPs, Sudoscan, and skin biopsy, to detect SFN, as relying on traditional patterns alone might overlook cases with atypical distributions. Neurologists should be aware of this possibility when evaluating post‐infectious neuropathic symptoms, especially in the context of “patchy” or borderline findings.

There were no differences in fatigue, autonomic, and neuropathic symptoms between PCC and ME/CFS patients. The absence of significant differences between these groups highlights the clinical overlap between them. In ME/CFS patients, the relationship found between fatigue and ESC could be indicative of autonomic neuropathy, which could contribute to fatigue [28]. These findings, coupled with results from QST and CHEPs, suggested that some patients may exhibit both sensory and autonomic SFN. Previous studies have also reported autonomic symptoms and abnormal ESC in PCC patients [29].

Fiber tortuosity emerged as a valuable indicator for potential SFN, suggesting that IVCCM alone might not be sufficient to detect SFN in these populations [30]. It is recommended to analyze the tortuosity of small nerve fibers and complements the assessment with other techniques to discern the type of SFN. The quantity and quality of small fibers in the cornea subbasal plexus inferior whorl could be more informative, particularly in neuropathies such as painful neuropathy [31]. IVCCM could also serve as a tool for detecting immunity‐related disorders or inflammation processes by quantifying and classifying different dendritic cells in the cornea subbasal plexus. Dendritic cells are known to be present in inflammatory processes and are more common in immune‐mediated diseases [14, 32, 33, 34, 35]. Previous studies have already detected the presence of these cells in PCC patients, suggesting the possible autoimmune nature of the disease [20, 36].

Finally, it is important to acknowledge the limitations of this study. The lack of skin biopsy data limits definitive conclusions regarding SFN prevalence. Likewise, a larger sample size could reveal significant differences between groups that may not be seen in this study.

Future studies should compare results from IVCCM and thermal‐evoked potentials with skin biopsy data to enhance diagnostic accuracy and validate findings, or compare the techniques used in this study with pathologies in which the presence of SFN is confirmed, such as amyloidosis. In this way, these tests ensure diagnostic sensitivity and specificity for SFN within these populations.

In conclusion, both PCC and ME/CFS patients demonstrated sensory SFN characterized by impaired heat detection and increased tortuosity of small fibers in the central cornea subbasal plexus. Traditional metrics like fiber density and length were insufficient for detecting neuropathy, underscoring the importance of analyzing fiber tortuosity and employing complementary techniques for a comprehensive assessment.

## AUTHOR CONTRIBUTIONS

**Naiara Azcue:** Investigation; writing – review and editing; formal analysis; writing – original draft; methodology. **Sara Teijeira‐Portas:** Investigation. **Beatriz Tijero‐Merino:** Writing – review and editing; investigation; conceptualization; methodology; project administration; supervision; funding acquisition. **Marian Acera:** Investigation. **Tamara Fernández‐Valle:** Investigation; conceptualization. **Unai Ayala:** Investigation; methodology; writing – review and editing. **Maitane Barrenechea:** Investigation; methodology. **Ane Murueta‐Goyena:** Writing – review and editing; investigation. **Jose Vicente Lafuente:** Investigation; writing – review and editing. **Adolfo Lopez de Munain:** Investigation. **Guillermo Ruiz‐Irastorza:** Investigation; writing – review and editing. **Daniel Martín‐Iglesias:** Investigation; writing – review and editing. **Iñigo Gabilondo:** Investigation; writing – review and editing. **Juan Carlos Gómez‐Esteban:** Investigation; funding acquisition; conceptualization; methodology; validation; writing – review and editing; supervision; project administration. **Rocio Del Pino:** Project administration; writing – review and editing; supervision; conceptualization; investigation; validation; methodology.

## FUNDING INFORMATION

This study has been funded by Instituto de Salud Carlos III (ISCIII) through the project PI20/01076 and co‐funded by the European Union, BIOEF through EITB maratoia (BIOS21/COV/006), and grants for health research projects from the Basque Government (2021111006). The first author received a pre‐doctoral research grant from the Basque Government (PRE_2023_2_0138).

## CONFLICT OF INTEREST STATEMENT

Nothing to report.

## Data Availability

Anonymized data not published within this article will be made available by request from any qualified investigator.
